# A Clathrin-Related Protein, SCD2/RRP1, Participates in Abscisic Acid Signaling in *Arabidopsis*

**DOI:** 10.3389/fpls.2020.00892

**Published:** 2020-06-18

**Authors:** Bingzhu Hou, Yuanyue Shen

**Affiliations:** ^1^Beijing Key Laboratory for Agricultural Application and New Technique, College of Plant Science and Technology, Beijing University of Agriculture, Beijing, China; ^2^Key Laboratory of Plant Molecular Physiology, Institute of Botany, Chinese Academy of Sciences, Beijing, China

**Keywords:** *Arabidopsis*, seed germination, seedling growth, abscisic acid, SCD2/RRP1, PYR1, ABI1

## Abstract

Abscisic acid (ABA) plays important roles in many aspects of plant growth and development, and responses to diverse stresses. Although much progress has been made in understanding the molecular mechanisms of ABA homoeostasis and signaling, the mechanism by which plant cells integrate ABA trafficking and signaling to regulate plant developmental processes is poorly understood. In this study, we used *Arabidopsis STOMATAL CYTOKINESIS DEFECTIVE 2*/*RIPENING-REGULATED PROTEIN 1* (*SCD2*/*RRP1*) mutants and overexpression plants, in combination with transcriptome and protein-interaction assays, to investigate SCD2/RRP1 involvement in the integration of ABA trafficking and signaling in seed germination and seedling growth. Manipulation of *SCD2/RRP1* expression affected ABA sensitivity in seed germination and seedling growth, as well as transcription of several ABA transporter genes and ABA content. RNA-sequencing analysis of *Arabidopsis* transgenic mutants suggested that SCD2/RRP1 was associated with ABA signaling *via* a type 2C protein phosphatase (PP2C) protein. The N- and C-terminal regions of SCD2/RRP1 separately interacted with both PYRABACTIN RESISTANCE 1 (PYR1) and ABA INSENSITIVE 1 (ABI1) on the plasma membrane, and SCD2/RRP1 acted genetically upstream of ABI1. Interestingly, ABA inhibited the interaction of SCD2/RRP1 with ABI1, but did not affect the interaction of SCD2/RRP1 with PYR1. These results suggested that in *Arabidopsis* SCD2/RRP1participates in early seed development and growth potentially through clathrin-mediated endocytosis- and clathrin-coated vesicle-mediated ABA trafficking and signaling. These findings provide insight into the mechanism by which cells regulate plant developmental processes through ABA.

## Introduction

During the lifetime of higher plants, the primary nutrient organs such as roots, stems, and leaves grow first for survival, followed by reproductive organs such as flowers, fruits, and seeds for sexual reproduction in response to diverse internal and external cues. At the cellular level, plant developmental processes essentially involve continuous cell division, differentiation, enlargement, and senescence. Each of these cellular processes is under the strict control of phytohormones.

Abscisic acid (ABA), a growth inhibitor identified in the early 1960s (Liu and Carnsdagger, 1961; Ohkuma et al., 1963), plays important roles not only in a variety of plant growth and developmental processes, including seed maturation and dormancy, seed germination, seedling and root growth, floral transition, fruit ripening, and stomatal movement, but also participates in the adaptive responses of plants to biotic and abiotic stresses, including drought, high salinity, chilling, and pathogen attack (Leung and Giraudat, 1998; Finkelstein et al., 2002; Himmelbach et al., 2003; Cutler et al., 2010; Zhang, 2014). These physiological responses are triggered by endogenous ABA contents, which are tightly controlled by ABA biosynthesis, catabolism, and transport (Nambara and Marion-Poll, 2005; Umezawa et al., 2006; Boursiac et al., 2013; Kuromori et al., 2018). In plants, ABA biosynthesis is synthesized from carotenoids, which are derived from the precursor of geranylgeranyl diphosphate by the methylerythritol phosphate pathway in plastids (Lichtenthaler, 1999). Geranylgeranyl diphosphate is also used for the biosynthesis of chlorophyll and gibberellins (Zi et al., 2014). To a large extent, free ABA homoeostasis is synergistically regulated at the synthesis–degradation and conjugation–hydroxylation levels by a set of critical enzymes, including 9-*cis*-epoxycarotenoid dioxygenase (NCED) for synthesis, ABA 8′-hydroxylases (CYP707A) for degradation, ABA glucosyltransferase for conjugation, and ABA β-glucosidase for hydroxylation (Schwartz et al., 2003; Lee et al., 2006). Several G-subfamily members of ATP-binding cassette (ABC) transporters, including ABCG 22/25/30/40, function in ABA trafficking (Boursiac et al., 2013; Merilo et al., 2015; Kuromori et al., 2018). Notably, AtNPF4.6/AtNRT1.2/AtAIT1 (a low-affinity nitrate transporter) and AtDTX50 (a member of the DTX/multidrug-toxic compound extrusion family) are ABA importers (Kanno et al., 2012; Léran et al., 2014). These diverse subcellular enzymes associated with ABA homoeostasis suggest the presence of ABA in different subcellular compartments, which is consistent with the multiple functions of ABA in plants in response to developmental and environmental cues (Xu et al., 2013).

Previous pharmacological and biochemical studies suggested the presence of both transmembrane and cytosolic ABA receptors in plants (Hornberg and Weiler, 1984; Allan et al., 1994; Assmann, 1994). Subsequently, multiple ABA receptors have been identified, including the plasma-membrane-localized GTG1/GTG2 (Pandey et al., 2009), a class of cytosolic PYR/PYL/RCAR (Ma et al., 2009; Park et al., 2009), and a plastid/chloroplast magnesium-chelatase H subunit ABAR/CHLH (Shen et al., 2006). Two core ABA signaling pathways in *Arabidopsis*(*Arabidopsis thaliana*) have been proposed, comprising the “ABA–PYR/PYL/RCAR–PP2C–SnRK2” pathway (Fujii et al., 2009), and the “ABA–ABAR–WRKY40–ABI5” pathway (Shang et al., 2010). Genetics and structural biology have confirmed the signaling transduction mechanism in the ABA–PYR/PYL/RCAR–PP2C–SnRK2 cascade: ABA binds to PYR/PYL/RCARs, which bind to and inhibit PP2C activity, and, as a result, the activated SnRK2s phosphorylate downstream targets to trigger ABA physiological responses (Fujii et al., 2009; Melcher et al., 2009; Miyazono et al., 2009; Park et al., 2009; Santiago et al., 2009; Soon et al., 2012). The ABA–PYR1–PP2C1–SnRK2 core signaling pathway also functions in strawberry fruit ripening (Chai et al., 2011; Jia et al., 2013).

It is known that ABA participates in complex biological processes and plays important roles in plant growth and development, as well as numerous stress responses. At the cellular level, plant growth and development requires continual cell division, enlargement, and adaptation in response to developmental and environmental cues. The multiple ABA receptors in different subcellular compartments contribute to the rapid response to physiological reactions. Although much progress has been made in elucidating the molecular mechanisms of ABA homoeostasis, transport, and signaling, the mechanism by which plant cells integrate ABA trafficking and signaling at different subcellular levels to regulate organ developmental processes is poorly understood.

STOMATAL CYTOKINESIS DEFECTIVE 2 (SCD2) functions in *Arabidopsis* cytokinesis and cell expansion through clathrin-mediated plasma membrane (PM) endocytosis and clathrin-coated vesicles (CCVs; McMichael et al., 2013). Interestingly, strawberry RIPENING-REGULATED PROTEIN 1 (FaRRP1), a ripening-regulated protein involved in fruit ripening (Shen et al., 2012, GenBank: JQ619656.1), is a homolog of SCD2. Given that ABA is a crucial regulator of ripening (Jia et al., 2011), clarification of the relationship between SCD2/RRP1 and ABA is important to understand ABA trafficking and signaling. In the present study, we used a series of *Arabidopsis* SCD2/RRP1 mutants and overexpression plants, in combination with transcriptome and protein-interaction analyses involving yeast two-hybrid (Y2H), co-immunoprecipitation (CoIP), bimolecular fluorescence complementation (BiFC), and luciferase (LUC) complementation assays, to investigate SCD2/RRP1 involvement in the integration of ABA trafficking and signaling in seed germination and seedling growth. The findings provide insight into the molecular mechanisms of endocytosis and CCVs mediated by the SCD2/RRP1 protein.

## Materials and Methods

### Plant Material and Growth Conditions

*Arabidopsis* accession Columbia (Col-0) wild-type (WT) plants were used in this study. The *RRP1* T-DNA insertion line SALK_063106, hereafter termed the *rrp1-1* mutant, was ordered from the *Arabidopsis* Biological Resource Center. The *rrp1-2* and *rrp1-3* mutants were generated using clustered regularly interspaced short palindromic repeats (CRISPR)/CRISPR-associated 9 (Cas9) technology. The *RRP1*-overexpression lines (*Super : RRP1-1* and *Super : RRP1-3*) were generated by cloning the coding sequence of *RRP1* into the *pBIB* vector under the control of the Super promoter. Seeds were surface sterilized with 70% (v/v) ethanol, sown on 0.8% (w/v) agar plates containing full-strength Murashige and Skoog (MS) medium supplemented with 1% or 3% (w/v) sucrose, stratified for three days at 4°C, and then grown under continuous light at 22°C.

### Real-Time PCR Analysis

For analysis of *Arabidopsis* gene expression, quantitative real-time PCR (qPCR) was performed with SYBR Premix Ex Taq (Takara) on a 7500 Real Time PCR System (Life Technologies, USA). The cDNAs were diluted 10 times with ddH_2_O, and 2 μl was used for PCR. The reaction conditions comprised 40 cycles at 95°C for 15 s and 60°C for 1 min. Relative gene expression was calculated using the 2^−ΔΔCT^ method (Livak and Schmittgen, 2001). *Arabidopsis ACTIN2/8* was used as an internal standard. The primers used are listed in **Supplemental Table 1**. The experiments were repeated three times.

### Determination of ABA Content

Three plants were used for each analysis. Gas chromatography–mass spectrometry (GC-MS) was used to analyze ABA content based on ^3^H-ABA (3,000 cpm) as an internal standard. Frozen receptacles (1 g) were mixed with sodium diethyldithiocarbamatetrihydrate, quartz sand, and 80% methanol containing radioactiveABA. The mixture and extracts were prepared in accordance with the method of Jia et al. (2011). The experiment was repeated three times.

### Phenotypic Analysis of *Arabidopsis*

For germination assays, seeds were surface sterilized and stratified at 4°C for 72 h in the dark before germination. More than 300 seeds of each genotype were sown on the same plate containing MS medium supplemented with 1% (w/v) sucrose and 0, 0.5, or 1 μM ABA, and incubated at 22°C under constant illumination of 60 μmol·m^−2^·s^−1^. Germination was defined as initial emergence of the radicle through the seed coat. The percentage seed germination was recorded daily during the germination test. For seedling growth experiments, seeds were germinated after stratification on standard MS medium. About 72 h after stratification, the seeds were transferred to MS medium supplemented with different concentrations of ABA and incubated in the vertical position. Seedling growth was assessed at the indicated times after transfer, and the length of the primary root was measured using a ruler.

### RNA-Sequencing and Data Analysis

Six *Arabidopsis* plants were randomly selected from the frozen samples for total RNA isolation and cDNA synthesis. Total RNA was extracted using the RNeasy Plant Mini Kit (Qiagen, Dusseldorf, Germany). DNase digestion was performed to remove contaminating DNA using the RNase-Free DNase Set (Qiagen). The RNA samples were processed using the RNA Library Prep Kit (New England BioLabs, Ipswich, MA, USA) and sequenced using an Illumina HiSeq2000 platform. The raw reads were filtered with the FASTQ_Quality_Filter tool from the FASTX-Toolkit. Reads longer than 35 bp and Q score > 20 were selected. All valid reads were combined to perform *de novo* splicing using the paired-end method with Trinity software (Wang et al., 2010). Data analysis was conducted in accordance with the method of Wang et al. (2017). The experiments were repeated three times.

### BiFC Assay

To determine whether the interaction between ABI1 or PYR1 and RRP1 takes place in planta, the coding sequences of *Arabidopsis ABI1* or *PYR1* and *RRP1* were cloned into the pSPYNE and pSPYCE vectors, respectively. The primer sequences used are listed in **Supplemental Table 1**. Plasmids containing *YFP^N^-ABI1*or *YFP^N^-PYR1* and *YFP^C^-RRP1* were introduced into *Agrobacterium tumefaciens* strain GV3101 and transformed into *Nicotiana benthamiana* leaves. Three days after infiltration, the yellow fluorescent protein (YFP) fluorescence signal was detected using a Zeiss LSM 710 META confocal microscope.

### CoIP Assay

*Arabidopsis* protoplasts transformed with the p35S:RRP1-Flag and pSuper : ABI1-GFP or pSuper : PYR1-GFP constructs were incubated in W5 buffer (154 mM NaCl, 125 mM CaCl_2_, 5 mM KCl, and 2 mM MES, pH 5.7) for 14–16 h. The pSuper : GFP construct was used as a negative control. The primers used to construct the vectors are listed in **Supplemental Table 1**. Total proteins were extracted from transformed protoplasts with 1-ml extraction buffer (10 mM HEPEs, pH 7.5, 100 mM NaCl, 1 mM EDTA, 10% glycerol, 0.5% Triton X-100, protease inhibitor cocktail, and 1 mM PMSF). The supernatant was immunoprecipitated with anti-Flag agarose (Sigma-Aldrich) for 2 h at 4°C. The immunoprecipitates were separated in a 12% SDS-PAGE gel and detected with anti-Flag and anti-GFP antibodies (Abmart).

### Y2H Assay

Y2H assays were performed using the Matchmaker GAL4-based two-hybrid system (Clontech, Palo Alto, CA, USA). Full-length or truncated cDNA of the corresponding genes was inserted into the pGADT7 and pGBKT7 vectors at the *Eco*RI/*Bam*HI restriction sites, respectively. The primer sequences used are listed in **Supplemental Table 1**. The constructs were transformed into *Saccharomyces cerevisiae*strain AH109 using a lithium acetate method. Yeast cells were cultured on selective medium −Leu/−Trp in accordance with the manufacturer’s instructions. Transformed colonies were plated onto selective medium −Leu/−Trp/−His/−Ade/+X-α-gal supplemented with 0 or 40 μM ABA to test for possible interactions.

### Firefly Luc Complementation Imaging Assay

For the Luc assays, the *Arabidopsis RRP1* and *ABI1* coding regions were amplified and cloned into the pCM1300-nLUC and pCM1300-cLUC vectors, respectively. The primers used to construct the vectors are listed in **Supplemental Table 1**. The plasmids were introduced into *Agrobacterium tumefaciens* strain GV3101 and co-infiltrated into the leaves of *N. benthamiana*. After incubation for 2 days, 0 or 100 μM ABA was sprayed onto the infected surface of infiltrated leaves for 6 h. The abaxial leaf surface was sprayed with 1 mM luciferin and then incubated in the dark for 10 min. The LUC signals were captured using a cooled CCD imaging camera (1300B, Roper) at −110°C. Relative LUC activity per cm^2^ infiltrated leaf area was calculated using Winview32 software. Three independent experiments were performed.

## Results

### Bioinformatics Analysis of SCD2/RRP1 Protein

To identify characteristics of the *Arabidopsis* SCD2/RRP1 protein, a Protein Blast (http://blast.ncbi.nlm.nih.gov/Blast.cgi) search using the 578-amino-acid sequence (*Arabidopsis* AT3G48860) determined that it is a coiled-coil domain-containing protein that belongs to a conserved superfamily containing a SMC (structural maintenance of chromosomes) domain, which have ATP-binding domains at the N- and C-termini and two extended coiled-coil domains separated by a hinge in the middle (**Supplemental Figure 1A**). Among the hits showing the highest homology, only RRP1 (Shen et al., 2012, GenBank: JQ619656.1, unpublished) and SCD2 (McMichael et al., 2013) have been annotated (**Supplemental Figure 1B**).

### Manipulation of SCD2/RRP1 Expression Affects *Arabidopsis* Seedling Growth

**Figure 1:**
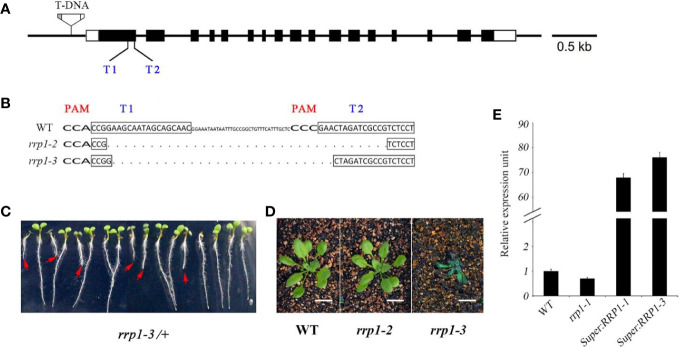
Manipulation of *Arabidopsis SCD2/RRP1* expression affects *Arabidopsis* seedling growth. **(A)** Schematic illustration of *RRP1* genomic regions with the *rrp1-1* T-DNA insertion and CRISPR/Cas9 targets. Empty boxes represent 5′ and 3′ untranslated regions, black boxes represent exons, and black lines represent introns. T1 and T2 represent two target sites for CRISPR/Cas9 editing. The T-DNA insertion is not drawn to scale. Bar = 0.5 kb. **(B)** Homozygous *rrp1* mutants of the T_3_ generation were generated by CRISPR/Cas9 editing. Mutation of *RRP1* was evaluated by sequencing. The protospacer adjacent motifs are marked with wide bold letters, the black boxes indicate targets, and the dashed line indicates regions of deletion. **(C)** Heterozygous *rrp1-3/+* mutants of the T_2_ generation grown vertically on Murashige and Skoog medium supplemented with 3% sucrose. The red arrows indicate homozygotes. **(D)** Phenotypic comparison between *rrp1-2*, *rrp1-3*, and wild type (WT) plants 4 weeks after germination. **(E)** Transcripts of the *rrp1-1* mutant and a *RRP1*-overexpression line (*Super : RRP1-3*) were tested by quantitative real-time PCR. The data represent the mean ± SE (*n* = 3).

In the *scd2-1* mutant, the guanine residue at position 1372 of the SCD2 open reading frame is deleted, leading to inactivation of the SCD2 domain, and plants show dwarfism and infertility (McMichael et al., 2013). Therefore, we screened for mutants in which gene expression, but not growth and development, were affected for further analysis. A set of transgenic *Arabidopsis* plants, comprising T-DNA insertion mutants, CRISPR/Cas9-edited mutants, and overexpression lines, was constructed. The T-DNA insertion line *rrp1-1* (SALK_063106) was ordered from the *Arabidopsis* Biological Resource Center. The CRISPR/Cas 9-generated *rrp1* mutants were produced using a pair of closely located sgRNA targets (T1 and T2) in *RRP1* (**Figure 1A**). In addition, two T_1_ lines of CRISPR/Cas9-generated *rrp1* mutants, named *rrp1-2* and *rrp1-3*, were screened and confirmed by sequencing, which demonstrated that the *rrp1-2* mutant was a homozygous mutant with a 68-bp deletion without a frame shift, whereas *rrp1-3*/+ was a heterozygous mutant with a 56-bp deletion with a frame shift inducing a premature stop codon (**Figure 1B**). No off-target mutations were detected (**Supplemental Table 2**). The heterozygous *rrp1-3*/+ plants grown on MS medium supplemented with 3% sucrose segregated on the basis of defects in plant growth and development (**Figure 1C**). Consistent with previous reports, homozygous *rrp1-3* plants grown in soil were dwarfed and infertile in comparison with the WT (**Figure 1D**). Real-time PCR analysis showed that *RRP1* expression was slightly repressed in the T-DNA mutants (*rrp1-1*) and elevated in the overexpression lines (*Super : RRP1-1* and *Super : RRP1-3*) in comparison with the WT (**Figure 1E**). These results demonstrated that alteration of *Arabidopsis SCD2*/*RRP1* expression affected plant growth.

### SCD2/RRP1 Is Involved in ABA-Mediated Seed Germination and Seedling Growth

**Figure 2:**
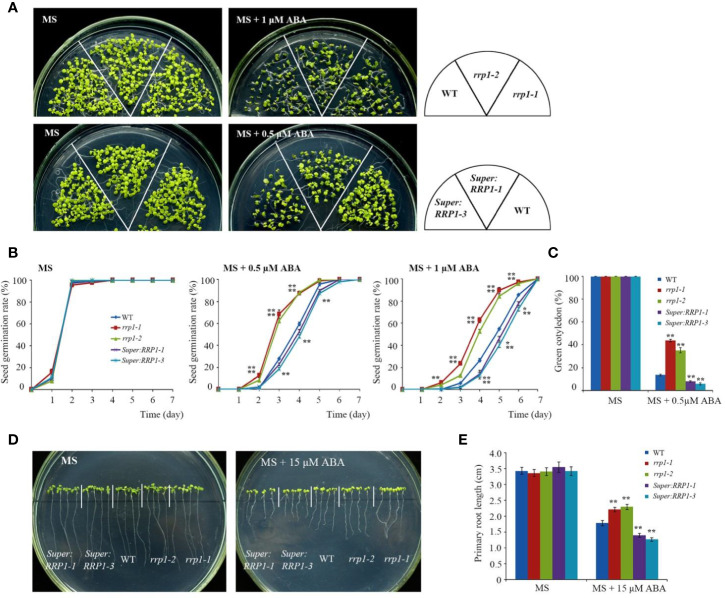
ABA sensitivity in the *rrp1* mutants and the *RRP1*-overexpression *Arabidopsis* lines. **(A)** Phenotypic comparison analysis. Imbibed seeds were transferred to Murashige and Skoog (MS), MS + 0.5 μM ABA, and MS + 1 μM ABA medium and grown for 14 days. **(B)** Seed germination assay. Imbibed seeds were transferred to MS and MS medium supplemented with 0.5 or 1 μM ABA. Seed germination frequency was calculated at a given time. Data are the mean ± SE (*n* = 3). More than 300 seeds were measured in each replicate. **(C)** Cotyledon-greening analysis. Imbibed seeds were transferred to MS or MS + 0.5 μM ABA medium for 6 days before determination of cotyledon-greening percentage. Data are the mean ± SE (*n* = 3). More than 300 seeds were measured in each replicate. **(D, E)** Primary root length in the presence or absence of exogenous ABA. The 3-day-old seedlings were transferred to MS or MS + 15 μM ABA medium for 7 days, and then photographs were taken and the primary root length was measured. Asterisks in **(B, C, E)** indicate statistically significant differences compared with wild-type plants: *, *P* < 0.05; **, *P* < 0.01.

To explore the role of SCD2/RRP1 in ABA-responsive physiological processes in *Arabidopsis*, owing to the infertility of homozygous *rrp1-3* plants, we used the *rrp1-1*, *rrp1-2*, *Super : RRP1-1*, and *Super : RRP1-3* transgenic plants to investigate seed germination and seedling growth on MS medium supplemented with 0, 0.5, or 1 μM ABA. All lines exhibited a similar phenotype (**Figure 2A**, left panel). The *rrp1-1* and *rrp1-2* mutants showed insensitivity to ABA on medium supplemented with 1 μM ABA, whereas the *Super : RRP1-1* and *Super : RRP1-3* lines were hypersensitive to 0.5 μM ABA compared with the WT (**Figure 2A**, right panel). No significant differences in percentage seed germination among the genotypes was observed on ABA-free MS medium. On medium supplemented with 0.5 or 1 μM ABA, the *rrp1-1* and *rrp1-2* mutants showed a significant increase in percentage seed germination compared with the WT (**Figure 2B**). In contrast, the seed germination percentages of the *Super : RRP1-1* and *Super : RRP1-3* lines were similar to that of the WT on medium containing 0.5 μM ABA, whereas germination decreased significantly on medium supplemented with 1 μM ABA (**Figure 2B**). The cotyledon-greening percentage was similar among the transgenic and WT plants on ABA-free medium (**Figure 2C**); in contrast, the *rrp1-1* and *rrp1-2* mutants showed higher percentages, whereas the *Super : RRP1-1* and *Super : RRP1-3* lines showed lower percentages, of cotyledon greening compared with that of the WT (**Figure 2C**).

To examine whether SCD2/RRP1 was involved in ABA-mediated root growth, 3-day-old seedlings of *rrp1* mutants, *RRP1*-OE lines, and the WT were transferred to MS medium supplemented with 0 or 15 μM ABA for 7 days. When grown on MS medium lacking ABA, the primary root length of *Super : RRP1-3* was similar, whereas that of the *rrp1-3*homozygotes was shorter, compared with the primary root of WT seedlings (**Figures 2D, E**). When grown on the medium supplemented with 15 μM ABA, the primary root lengths of the *Super : RRP1-1* and *Super : RRP1-3* lines were shorter, whereas those of the *rrp1-1*and *rrp1-2* mutants were longer, compared with the primary root of the WT (**Figures 2D, E**). Taken together, these data demonstrated that SCD2/RRP1 served as a positive regulator of ABA-mediated *Arabidopsis* seed germination and seedling growth.

### RNA-Sequencing Analysis Suggests SCD2/RRP1 Is Associated With ABA Signaling *via* PP2C

To identify candidate SCD2/RRP1-regulated genes in the transgenic plants, three groups comprising the *rrp1-3* mutants (homozygotes gained by CRISPR), the *RRP1*-overexpression line (*Super : RRP1-3*), and the WT were used for RNA-sequencing (RNA-seq) in accordance with a previous report on differentially expressed genes (DEGs) at the log_2_ (2 RPKM) level (Wang et al., 2017). Comparing the *rrp1-3* mutants with the WT, 341 DEGs were annotated with 156 upregulated and 185 downregulated among the mapped 19,130 genes in the two libraries; comparing *Super : RRP1-3* with the WT, 96 DEGs were annotated with 23 upregulated and 73 downregulated among the mapped 19,102 genes in the two libraries; and comparing *Super : RRP1-3* with the *rrp1-3* mutants, 331 DEGs were annotated with 171 upregulated and 160 downregulated among the mapped 19,105 genes in the two libraries (**Table 1**). It was notable that among the three-group comparisons, the majority of DEG-mapped pathways were detected in the comparison of *Super : RRP1-3* with the *rrp1-3* mutants; nine pathways were detected, among which the top-five pathways comprised α-linolenic acid metabolism, glucosinolate biosynthesis, 2-oxocarboxylic acid metabolism, degradation of valine, leucine and isoleucine, and plant hormone signal transduction.

**Table 1:** The differentially expressed genes among the transgenic and wild type plants by RNA-seq.

CRISPR/WT/OE	All Genes	All DEG	UP	DOWN
*rrp1-3 mutants*/Wild type	19,130	341	156	185
*Super : RRP1-3*/Wild type	19,102	96	23	73
*rrp1-3 mutants*/*Super : RRP1-3*	19,105	331	171	160

DEG, differentially expressed gene; UP, upregulation; DOWN, downregulation.

**Figure 3:**
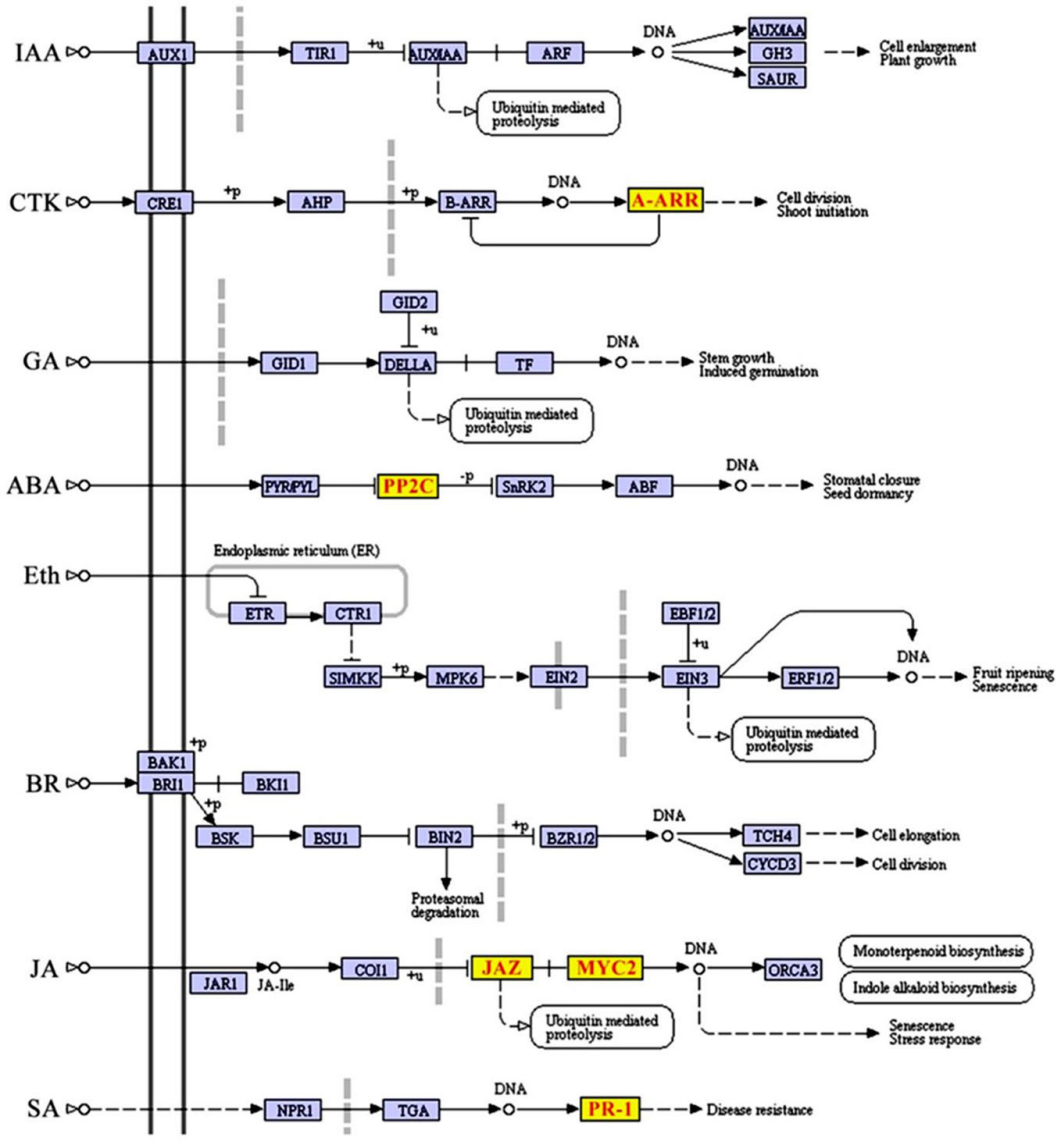
Differentially expressed genes (DEGs) mapped to the plant hormone signal transduction pathway according to a Kyoto Encyclopedia of Genes and Genomes (KEGG) enrichment analysis. KEGG terms with a corrected *P*-value less than 0.05 were considered to be significantly enriched by DEGs. Five DEGs, comprising *JAZ*, *MYC2*, *ARR-A*, *PR1*, and *PP2C*, were screened and transcript levels were upregulated, which are shown in red letters on yellow blocks in the plant hormone signal transduction pathway.

**Figure 4:**
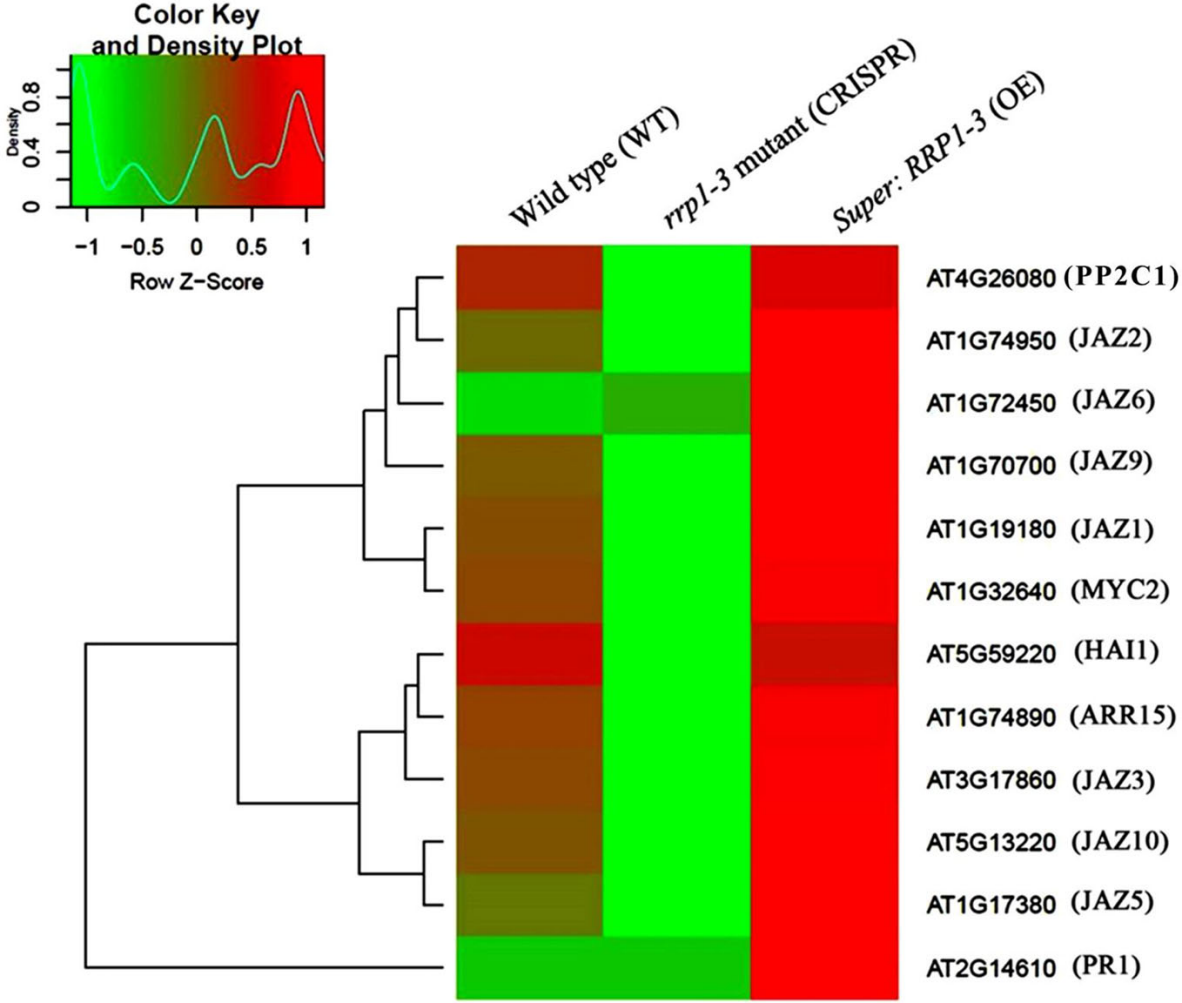
Heatmap and cluster analysis of transcripts for the important genes associated with RRP1 in *Arabidopsis*. Data for gene expression levels were normalized to the *z*-score with the formula log_10_ (FPKM + 1) and are indicated by a color key and density plot; from green to red represents a relative gene expression level from low to high, respectively. ABI1, ABA-insensitive 1; HAI1, highly ABA-Induced1; JAZ1, 2, 3, 5, 6, 9, and 10, Jasmonate ZIM-domain1, 2, 3, 5, 6, 9, and 10; MYC2, myelocytomatosis protein 2 (a basic helix-loop-helix Leu zipper transcription factor); ARR15, two-component type-A response regulator; and PR1 (pathogenesis-related protein 1).

To further investigate whether SCD2/RRP1 is involved in phytohormone signaling pathways, the DEGs mapped to this pathway were analyzed in relation to gibberellin, ABA, ethylene, brassinolide, jasmonic acid (JA), salicylic acid, cytokinin, and auxin. Five DEG families, consisting of PP2C, JAZ, MYC2, A-ARR, and PR1, were annotated to phytohormone pathways (**Figure 3**): ABA (ABI1: ABA-insensitive 1; HAI1: highly ABA-induced 1), JA (JAZ1, 2, 3, 5, 6, 9, and 10: Jasmonate ZIM-domain 1, 2, 3, 5, 6, 9, and 10; MYC2: myelocytomatosis protein 2, a basic helix-loop-helix Leu zipper transcription factor), cytokinin (ARR15: two-component type-A response regulator), and salicylic acid (PR1: pathogenesis-related protein 1; **Figure 4**). Interestingly, the majority of these DEGs were upregulated in the OE plants and downregulated in the CRISPR/Cas9 plants in comparison with the WT (**Figure 4**). Given that these transgenic plants showed no response to JA in seed germination and seedling growth (data not shown), these results suggested that SCD2/RRP1 might be involved in ABA signaling through the ABI1 protein, which is a crucial negative regulator of ABA signaling.

### Investigation of Interaction Regions Between SCD2/RRP1 With ABI1 and Effects of ABA on the Interaction

**Figure 5:**
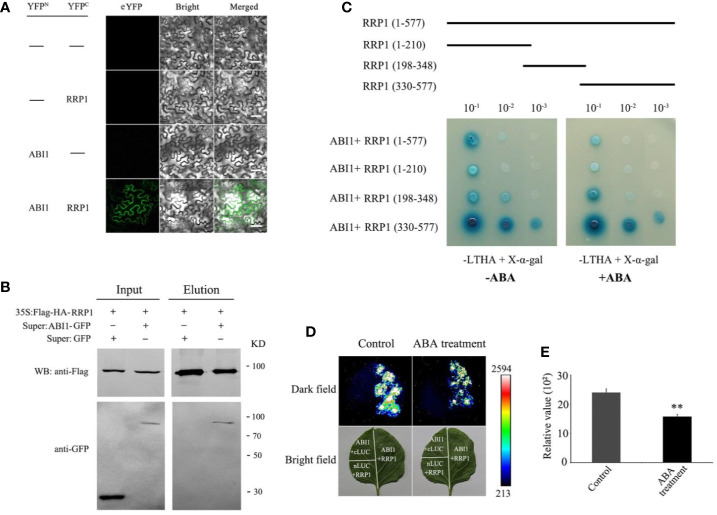
Interaction of RRP1 with ABI1 in *Arabidopsis* and the influence of ABA. **(A)** Bimolecular fluorescence complementation (BiFC) assay showing the interaction of RRP1 and ABI1. eYFP, enhanced yellow fluorescent protein. YFP^N^ and YFP^C^ are the N terminus and C terminus of eYFP, respectively. Scale bars, 20 μm. **(B)** Co-immunoprecipitation (Co-IP) assay of RRP1 with ABI1. *Super : RRP1-GFP* and *35S:Flag-HA-ABI1*, or *Super : GFP* and *35S:Flag-HA-ABI1* were co-transfected into *Arabidopsis* protoplasts. Co-IP was conducted with anti-Flag beads from total isolated proteins, and immunoblotting analysis was performed with anti-GFP and anti-Flag antibodies. **(C)** The C-terminus of RRP1 primarily interacts with ABI1, and ABA weakens RRP1–ABI1 interaction in a Y2H assay. Top panels show a schematic representation of the different RRP1 deletions in the yeast vectors. Yeast transformants were spotted onto the selective medium SD/−Leu/−Trp/−His/−Ade (−LTHA)/+X-α-gal supplemented with 0 or 40 μM ABA. **(D)** ABA weakens RRP1–ABI1 interaction in a firefly luciferase (LUC) complementation imaging system. *Nicotiana benthamiana* leaves were transformed with the construct pairs ABI1-nLUC/RRP1-cLUC and negative controls (ABI1-nLUC/cLUC and nLUC/RRP1-cLUC). The leaves were observed for fluorescence imaging 6 h after spraying 0 or 100 μM ABA on the infected leaf surface. **(E)** Quantitative analysis of luminescence intensity. Each value is the mean ± SE of three independent experiments. Asterisks indicate statistically significant differences compared with the control: **, *P* < 0.01.

To explore whether SCD2/RRP1 has a direct relationship with ABI1, BiFC and a *N. benthamiana* transient expression analysis were performed by co-infiltration of *Agrobacterium tumefaciens* harboring ABI1-YNE and RRP1-YCE into *N. benthamiana*leaf epidermal cells *in vivo*. After cultivation for 2.5 days, the fluorescence signal at 488 nm was observed. The YFP fluorescence signal was detected from the two-gene, co-infected leaves, whereas the control infected with the empty vector showed no YFP signal; notably, the interaction signals may be entirely cytosolic (**Figure 5A**).

To further confirm this interaction, CoIP was used based on construction of RRP1-Flag and ABI1-GFP fusion protein vectors, which were co-transformed into *Arabidopsis* protoplasts and cultured for 16 h. The total protoplast proteins were extracted for CoIP western blotting, which revealed that the ABI1-GFP protein could be co-precipitated by anti-Flag beads and then detected by the anti-GFP antibody, and vice versa, whereas this was not observed for the control infected with the empty vector (**Figure 5B**). These results further demonstrated that RRP1 interacts with ABI1 *in vivo*.

To identify the crucial regions in SCD2/RRP1 for interaction with ABI1, the coding sequence of *RRP1* was truncated into three sections (1–210, 198–348, and 330–577 bp). These cDNA fragments were ligated into the pGADT7 vector, co-transformed into yeast AH109 cells with the pGBKT7-RRP1 construct, then plated separately on SD/−Trp/−Leu/−His/−Ade/+X-α-Gal medium supplemented with 0 or 40 μM ABA, and cultured for 2–5 days. ABI1 interacted strongly with the polypeptide encoded by the 330–577 fragments, but interacted only weakly with the polypeptides encoded by the 1–210 and 198–348 fragments, and these interactions may be affected by ABA (**Figure 5C**).

To verify the effect of ABA on the interaction between RRP1 and ABI1, a LUC complementation assay *in vivo* was conducted. The coding sequences of RRP1 and ABI1 were separately ligated to generate the ABI1-nLUC and RRP1-cLUC constructs. *Agrobacterium tumefaciens* harboring the ABI1-nLUC and RRP1-cLUC fusion plasmids were co-infected into the lower epidermal cells of *N. benthamiana* leaves. After cultivation for 2.5 days, the infected leaf surfaces were sprayed with 0 μM (control) or 100 μM ABA solution. After culture for 6 h, the leaf surfaces were sprayed with LUC fluorescent substrate and reacted for 10 min, then the fluorescence signal was detected. The *N. benthamiana*leaves treated with 100 μM ABA showed weaker fluorescence compared with those treated with 0 μM ABA (**Figures 5D, E**). These results confirmed that SCD2/RRP1 is capable of interacting with ABI1, and indicated that interaction occurs at the C-terminus of SCD2/RRP1, which is affected by ABA.

### SCD2/RRP1 Is Genetically Upstream of ABI1

To explore the genetic relationship between SCD2/RRP1 and ABI1, the ABA-insensitive *rrp1-2* mutant and ABA-sensitive *Super : RRP1-3* plants (**Figure 1**) as well as the ABA-sensitive *abi1-cas9* mutants (our previously constructed knockout mutant at nucleotide position 536; data not shown) were used to generate F_2_ plants comprising *abi1-cas9*/*rrp1-2* and *abi1-cas9/Super : RRP1-3* by hybridization, screening, and identification (data not shown).

**Figure 6:**
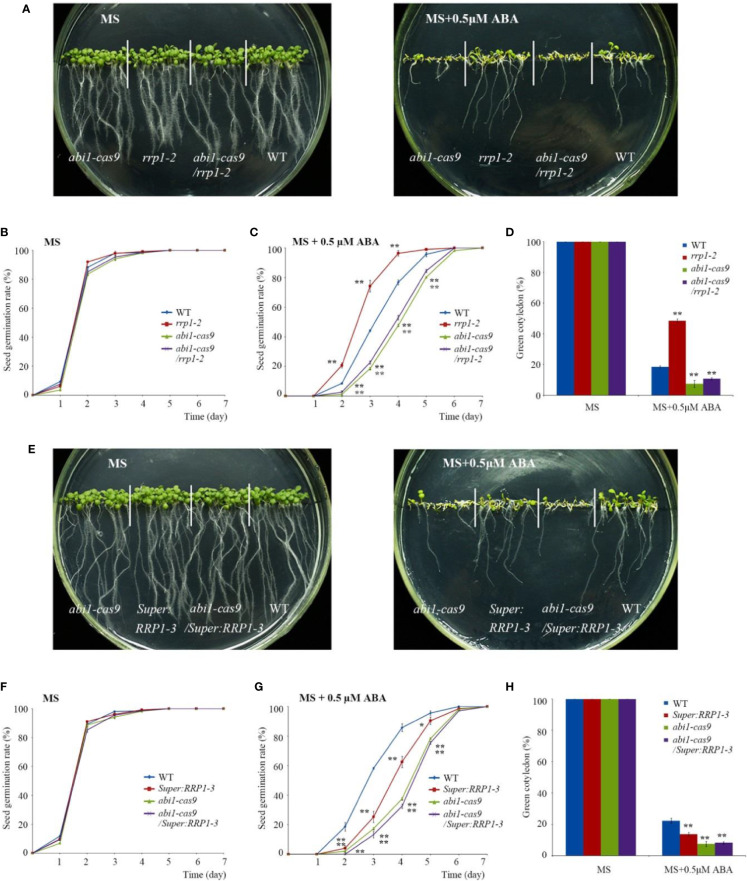
Function of RRP1 upstream of ABI1. **(A)** Phenotypic comparison analysis. Imbibed seeds were transferred to Murashige and Skoog (MS) and MS + 0.5 μM abscisic acid (ABA) medium and grown for 9 days. **(B, C)** Seed germination assay. Imbibed seeds were transferred to MS **(B)** and MS medium supplemented with 0.5 μM ABA **(C)**. The seed germination frequency was calculated at a given time. Data are the mean ± SE (*n* = 3). More than 300 seeds were measured in each replicate. **(D)** Cotyledon-greening analysis. Imbibed seeds were transferred to MS or MS + 0.5 μM ABA medium for 7 days before determination of cotyledon-greening percentage. Data are the mean ± SE (*n* = 3). More than 300 seeds were measured in each replicate. **(E)** Phenotypic comparison analysis. Imbibed seeds were transferred to MS and MS + 0.5 μM ABA medium and grown for 11 days. **(F, G)** Seed germination assay. Imbibed seeds were transferred to MS **(B)** and MS medium containing 0.5 μM ABA **(C)**. The seed germination frequency was calculated at a given time. Data are the mean ± SE (*n* = 3). More than 300 seeds were measured in each replicate. **(H)** Cotyledon-greening analysis. Imbibed seeds were transferred to MS or MS + 0.5 μM ABA medium for 7 days before determination of cotyledon-greening percentage. Data are the mean ± SE (*n* = 3). More than 300 seeds were measured in each replicate. Asterisks indicate statistically significant differences compared with the wild-type plants: *, *P* < 0.05; **, *P* < 0.01.

First, various phenotypes were observed using the *abi1-cas9*, *rrp1-2*, *abi1-cas9/rrp1-2*, and WT plants. Seed germination and growth analysis showed that on standard MS medium, all plant materials germinated and grew with no significant differences observed (**Figure 6A**, left, and **Figure 6B**). However, on medium supplemented with 0.5 μM ABA, the *rrp1-2* mutant displayed an ABA-insensitive phenotype, whereas the *abi1-cas9/rrp1-2*double mutant showed an ABA-sensitive phenotype, similar to that of the *abi1-cas9* mutant (**Figures 6A**, right, **C**). Analysis of the seedling cotyledon-greening percentage showed that on standard MS medium, all transgenic plants showed similar growth trends. On medium supplemented with 0.5 μM ABA, the *rrp1-2* mutant showed a higher cotyledon-greening percentage, whereas the *abi1-cas9/rrp1-2* double mutant showed a lower percentage, compared with that of the WT, similar tothe *abi1-cas9* mutant (**Figure 6D**).

Second, various phenotypes were observed using the *abi1-cas9*, *Super : RRP1-3*, *abi1-cas9/Super : RRP1-3*, and the WT plants. On standard MS medium, all plant materials germinated and grew with no significant differences observed (**Figures 6E**, left, **F**). On medium supplemented with 0.5 μM ABA, germination and growth of *Super : RRP1-3* and *abi1-cas9/Super : RRP1-3* plants displayed an ABA-sensitive phenotype, and the degree of sensitivity of the *abi1-cas9/Super : RRP1-3* plants was similar to that of the *abi1-cas9* mutant (**Figures 6E**, right, **G**). Analysis of the seedling cotyledon-greening percentage showed that on standard MS medium, all transgenic plants showed similar growth trends. However, on medium supplemented with 0.5 μM ABA, *Super : RRP1-3* and *abi1-cas9/Super : RRP1-3* plants showed a lower cotyledon-greening percentage compared with that of the WT, but the percentage of the *abi1-cas9/Super : RRP1-3* plants was similar to that of the *abi1-cas9* mutant (**Figure 6H**).

Taken together, these results indicated that *ABI1* knockout impaired not only the ABA-insensitive phenotypes of the *rrp1-2* mutant, but also the ABA-sensitive phenotype of the *RRP1*-overexpressing lines in seed germination and seedling growth, which suggested that SCD2/RRP1 is genetically upstream of AtABI1 in the response to ABA.

### Interaction Analysis of PYR1 With SCD2/RRP1 and Effects of ABA on the Interaction

**Figure 7:**
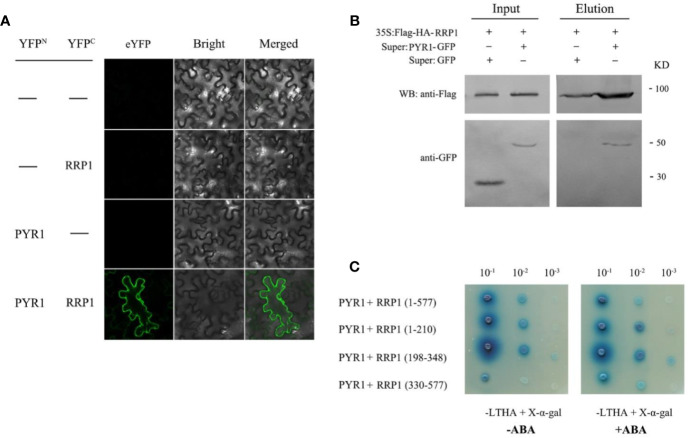
Interaction of RRP1 with PYR1 in *Arabidopsis*. **(A)** BiFC assay showing interaction of RRP1 and PYR1. eYFP, enhanced yellow fluorescent protein. YFP^N^ and YFP^C^ are the N terminus and C terminus of eYFP, respectively. Scale bars, 20 μm. **(B)** Co-IP assay of RRP1 with PYR1. *Super : PYR1-GFP* and *35S:Flag-HA-RRP1*, or *Super : GFP* and *35S:Flag-HA-RRP1* were co-transfected into *Arabidopsis* protoplasts. Co-IP was conducted with anti-Flag beads from total isolated proteins, and immunoblotting analysis was performed with anti-GFP and anti-Flag antibodies. **(C)** The N-terminus of RRP1 primarily interacts with PYR1. Yeast transformants were spotted onto the selective medium SD/−Leu/−Trp/−His/−Ade (−LTHA)/+X-α-gal supplemented with 0 or 40 μM ABA.

Interaction of SCD2/RRP1 with ABI1 prompted us to explore the relationship between SCD2/RRP1 and PYR1. Therefore, Y2H, BiFC, and CoIP assays were conducted as described for the assays with ABI1. In the BiFC and CoIP interaction systems, RRP1 interacted directly with PYR1 *in vivo*, and interaction in the cytoplasm was observed (**Figures 7A, B**). The Y2H assay showed that PYR1 interacted with the polypeptide encoded by the 1–210 and 198–348 fragments of *RRP1*, and the interactions were not affected by ABA (**Figure 7C**). These results confirmed that SCD2/RRP1 is capable of interacting with PYR1 and the interaction can occur in the cytoplasm, and that interaction occurs in the N-terminus of RRP1 in an ABA-independent manner.

### Manipulation of SCD2/RRP1 Expression Affects Expression of a Set of ABA Transporter Genes and ABA Contents in *Arabidopsis*

**Figure 8:**
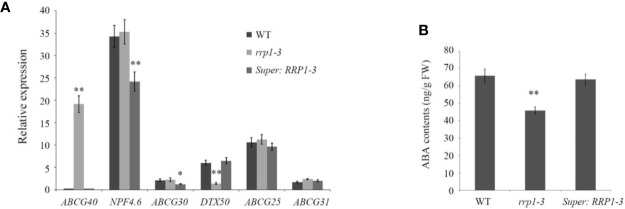
Analysis of relative expression levels of ABA transporter genes and ABA contents intransgenic *Arabidopsis* plants. **(A)** Transcripts of a set of ABA transporter genes were analyzed by quantitative real-time PCR in the wild-type, *rrp1-3*, and *Super : RRP1-3* plants. **(B)** ABA contents in the wild-type, *rrp1-3*, and *Super : RRP1-3* plants. The data represent the mean ± SE (*n* = 3). Asterisks indicate statistically significant differences compared with wild-type plants: *, *P* < 0.05; **, *P* < 0.01.

Transport of ABA in *Arabidopsis* cells is regulated by ABCG40, NPF4.6, and ABCG30, which are involved in ABA uptake, and DTX50, ABCG25, and ABCG31, which participate in ABA efflux (Dong et al., 2015; Merilo et al., 2015; Kuromori et al., 2018). To investigate whether manipulation of *SCD2/RRP1* gene expression influences overall transport capacity of ABA in WT, *rrp1-3*, and *Super : RRP1-3* cells, the ABA contents and mRNA levels of several ABA transporter genes were analyzed on the basis of the RNA-seq data. The qPCR analysis showed that with regard to ABA uptake and transport, *ABCG40* transcripts were upregulated by sixty-fold in the *rrp1-3* mutant, whereas the transcripts of *NPF4.6* and *ABCG30* were significantly downregulated in overexpression lines. With regard to ABA efflux, the *DTX50* transcript level was significantly downregulated by four-fold in the *rrp1-3* mutant, whereas the transcript levels of *ABCG25* and *ABCG31* were not significantly affected in the overexpression lines (**Figure 8A**). The ABA contents were reduced in the *rrp1-3* mutant, but no significant effect was observed in the *Super-RRP1-3* line in comparison with the control (**Figure 8B**). These results further indicated that SCD2/RRP1 was associated with ABA trafficking.

## Discussion

### The Role of SCD2/RRP1 in Seed Germination and Seedling Growth Is Associated With ABA

The *Arabidopsis* SCD1 protein, a Rab guanine nucleotide exchange factor, functions in cytokinesis and cell expansion by membrane trafficking (Falbel et al., 2003). Similarly, SCD2 is co-localized with SCD1 in the clathrin light chain at sites of endocytosis in the PM, functions at hypostasis to SCD1, and both proteins are required for clathrin-mediated plasma-membrane endocytosis and CCVs, which are crucial to cytokinesis and cell expansion (McMichael et al., 2013).

Whether SCD2/RRP1functions in relation to phytohormones is yet unknown. In the present study, we provide evidence to demonstrate that SCD2/RRP1 regulates *Arabidopsis* seed germination and seedling growth *via* ABA: (1) manipulation of *SCD2/RRP1* expression in the mutants affects ABA sensitivity in seed germination and seedling growth (**Figure 2**); (2) SCD2/RRP1 can interact with ABI1, which is a core component of ABA core signaling (Fujii et al., 2009; Ma et al., 2009), and the interaction is inhibited by ABA (**Figure 5**); (3) SCD2/RRP1 can interact with PYR1, which is an ABA receptor (Fujii et al., 2009; Ma et al., 2009; **Figure 7**); and (4) manipulation of *SCD2/RRP1* expression affects ABA contents and transcription of several ABA transporter genes (**Figure 8**).

### A Potential Molecular Mechanism of SCD2/RRP1 in *Arabidopsis* Seed Germination and Seedling Growth

Sessile plants have developed a series of mechanisms for growth and survival. Endocytosis is a mechanism by which certain molecules are transported into cells across the PM (Zwiewka et al., 2015; Mettlen et al., 2018). Clathrin-mediated endocytosis, which is the best-characterized endocytic pathway, is a major regulator of cell-surface protein internalization in response to extracellular and intracellular cues (Kitakura et al., 2011; Irani et al., 2012; Zwiewka et al., 2015; Belda-Palazon et al., 2016; Mettlen et al., 2018). Interestingly, clathrin-mediated endocytosis is involved in auxin transport (Kitakura et al., 2011), brassinosteroid signaling (Irani et al., 2012), and ABA signaling (Belda-Palazon et al., 2016). However, mechanisms mediated by clathrin-mediated endocytosis for cell-surface protein internalization and trafficking are not fully understood in response to phytohormones.

**Figure 9:**
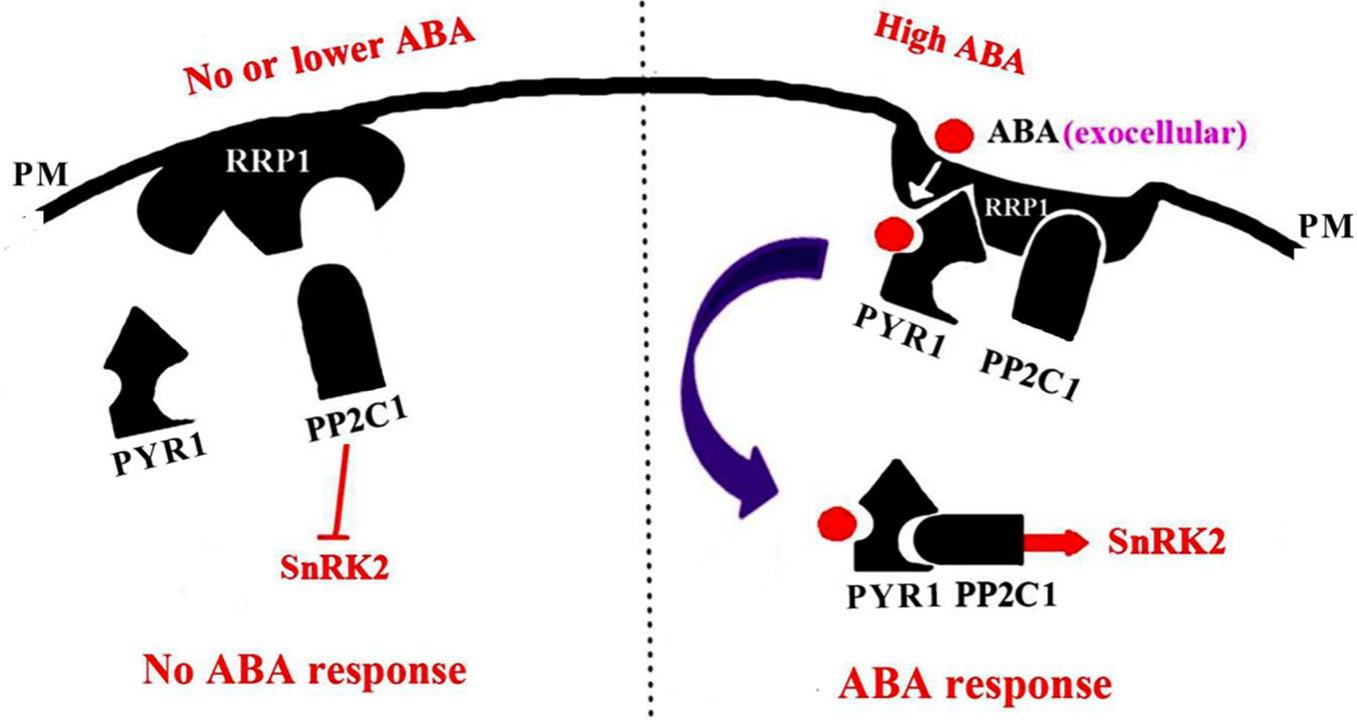
A model for SCD2/RRP1-mediated endocytosis of ABA and ABA signaling. When the ABA content is low or absent, RRP1 may bind to and activate ABI1 activity and, as a result, promote inhibition of SnRK2 activity and ABA response. When the ABA content is high, ABA binds to RRP1 to promote the RRP1 recruitment of PYR1 and ABI1 to the plasma membrane (PM) to form a three-protein complex, which then further facilitates SCD2/RRP1-mediated endocytosis of ABA and ABA signaling.

*Arabidopsis* SCD2 functions in clathrin-mediated membrane transport, including PM endocytosis, which is required for cytokinesis and cell expansion (McMichael et al., 2013). Manipulation of *Arabidopsis SCD2/RRP1*expression affects transcription of several ABA transporter genes and ABA contents (**Figure 8**), thus RRP1 may potentially be involved in ABA trafficking through clathrin-mediated membrane transport and endocytosis. Interestingly, interaction of SCD2/RRP1 with PYR1 and ABI1 was confirmed (**Figures 5**, **6**, **7**). Furthermore, ABA did not affect interaction of the N-terminus of SCD2/RRP1 with PYR1, but inhibited interaction of the C-terminus of SCD2/RRP1 with ABI1 (**Figures 5** and **7**), which suggested that SCD2/RRP1 may recruit PYR1 and ABI1 onto the PM and CCVs. The ABA–PYR1–ABI1–SnRK2.2 cascade is a core signaling mechanism, by which ABA regulates plant growth and development as well as stress responses (Fujii et al., 2009; Ma et al., 2009; Jia et al., 2013). Given that SCD2 is a peripheral membrane protein (McMichael et al., 2013), we thus speculate on the function of SCD2/RRP1 in *Arabidopsis* growth and development (**Figure 9**). The SCD2/RRP1 protein may recruit PYR/PYLs/RCARs and PP2Cs to form a three-protein complex near the PM, and then may initiate clathrin-mediated endocytosis and CCVs. ABA can inhibit interaction of RRP1 with ABI1 and, as a result, promotes ABA responsiveness and the ABA–PYR/PYLs/RCARs–PP2Cs–SnRK2 cascade, ultimately regulating plant growth and development. Further exploration is a prerequisite for visualization of the cellular processes in SCD2/RRP1-mediated ABA trafficking and signaling by endocytosis and vesicle transport.

## Data Availability Statement

The datasets generated for this study can be found in the NCBI BioProject ID PRJNA635263.

## Author Contributions

BH performed the experiments. YS designed the research and wrote the article.

## Funding

the National Natural Science Foundation of China (Project 31672125).

## Conflict of Interest

The authors declare that the research was conducted in the absence of any commercial or financial relationships that could be construed as a potential conflict of interest.
