# Supplementary material for: A Clathrin-Related Protein, SCD2/RRP1, Participates in Abscisic Acid Signaling in Arabidopsis
Source: Front Plant Sci. 2020 Jun 18;11:892. doi: 10.3389/fpls.2020.00892 (PMC7314967; doi:10.3389/fpls.2020.00892)
Supplement: Supplementary file 1 [file DataSheet_1.docx]

**Supplementary Data**

**Supplementary Tables**

**Supplementary Table 1.**The primers used in this study.

| **Primers for confirming T-DNA insertion** | |
| --- | --- |
| Primer name | Sequence |
| LBNEW-SALK | 5'- ATTTTGCCGATTTCGGAAC -3' |
| *rrp1-1*-LP | 5'- GATTAGGAATCGGAGTCGGAG -3' |
| *rrp1-1*-RP | 5'- TCTCCAACATTGTTGGGCTAG -3' |
| **Primers for RT-PCR analysis** | |
| Primer name | Sequence |
| *RRP1*-RT-F | 5'- CCATCCAGGCAAGAGCACT -3' |
| *RRP1*-RT-R | 5'- CCCATCTCAACTGAAAGCATACT -3' |
| *ABCG25-*RT-F | 5'- ACGACGGTAAAATAACGAAACAAA -3' |
| *ABCG25-*RT-R | 5'- GACTCCGAGGAAGACGAAGC -3' |
| *ABCG30-*RT-F | 5'- ATCTGGTGGCGAGAAGAGGAGG -3' |
| *ABCG30-*RT-R | 5'- GGTGCAGGCTGAAGAAGTGAAA -3' |
| *ABCG31-*RT-F | 5'- TCTAACGCCGAGACAGTGGAGC -3' |
| *ABCG31-*RT-R | 5'- GACGCAGAATCGCATTATGAG -3' |
| *ABCG40-*RT-F | 5'- GCCTTACTTGGGTTCGTCGTG -3' |
| *ABCG40-*RT-R | 5'- TCTGTCTTCTTGTGTGCCTTGCT -3' |
| *DTX50-*RT-F | 5'- GTCGCTCAATCTTTCTTACACCC -3' |
| *DTX50-*RT-R | 5'- AGTTTCTTCCATTCTCTCACACTATCT -3' |
| *Actin2/8*-F | 5'- GGTAACATTGTGCTCAGTGGTGG -3' |
| *Actin2/8*-R | 5'- AACGACCTTAATCTTCATGCTGC -3' |
| **Primers for Y2H assay** | |
| Primer name | Sequence |
| ABI1-AD-F | 5'- GCCATGGAGGCCAGTGAATTCATGGAGGAAGTATCTCCGGC -3' |
| ABI1-AD-R | 5'- CAGCTCGAGCTCGATGGATCCGTTCAAGGGTTTGCTCTTGAGTT -3' |
| PYR1-AD-F | 5'- GCCATGGAGGCCAGTGAATTCATGCCTTCGGAGTTAACACC -3' |
| PYR1-AD-R | 5'- CAGCTCGAGCTCGATGGATCCCGTCACCTGAGAACCACTTC -3' |
| RRP1-1BD-F | 5'- CGGAATTCATGGATCGGAGGAGGGCGGG -3' |
| RRP1-1BD-R | 5'- CGGGATCCATCACCCTTTTCTTTTGAATTTAC -3' |
| RRP1-2BD-F | 5'- CGGAATTCATGGATGTACCATCAGTAAATTCAAAAG -3' |
| RRP1-2BD-R | 5'- CGGGATCCCAGAACTACTTCTTCCATTTCATC -3' |
| RRP1-3BD-F | 5'- CGGAATTCATGACAATGACTCAGAGGATGATTTTA -3' |
| RRP1-3BD-R | 5'- CGGGATCCTGAATCAGCATCGAGATCGTG -3' |
| **Primers for BiFC assay** | |
| Primer name | Sequence |
| RRP1-YCE-*Bam*HI F | 5'- TGGCGCGCCACTAGTGGATCCATGGATCGGAGGAGGGC -3' |
| RRP1-YCE-*Xho*I R | 5'- CCCGGGAGCGGTACCCTCGAGTGAATCAGCATCGAGATCGTGAT -3' |
| ABI1-YNE-*Spe*I F | 5'- CAGGCCTGGCGCGCCACTAGTATGGAGGAAGTATCTCCGGC -3' |
| ABI1-YNE-*Xho*I R | 5'- CCCGGGAGCGGTACCCTCGAGGTTCAAGGGTTTGCTCTTGAGTT -3' |
| PYR1-YNE-*Spe*I F | 5'- CAGGCCTGGCGCGCCACTAGTATGCCTTCGGAGTTAACACC -3' |
| PYR1-YNE-*Xho*I R | 5'- CCCGGGAGCGGTACCCTCGAGCGTCACCTGAGAACCACTTC -3' |
| **Primers for Co-IP assay** | |
| Primer name | Sequence |
| RRP1-Flag-*Sac*I F | 5'- ATCGATACCGTCGACGAGCTCATGGATCGGAGGAGGGCGGG -3' |
| RRP1-Flag-*Kpn*I R | 5'- TTTGCGGAGTACCCGGGTACCCTATGAATCAGCATCGAGATCGT -3' |
| ABI1-GFP-*Spe*I F | 5'- GGACTAGTATGGAGGAAGTATCTCCGGC -3' |
| ABI1-GFP-*Kpn*I R | 5'- GGGGTACCGTTCAAGGGTTTGCTCTTGAGTT -3' |
| PYR1-GFP-*Spe*I F | 5'- GGACTAGTATGCCTTCGGAGTTAACACC -3' |
| PYR1- GFP-*Kpn*I R | 5'- GGGGTACCCGTCACCTGAGAACCACTTC -3' |
| **Primers for LCI assay** | |
| Primer name | Sequence |
| ABI1-nLUC-*Kpn*I F | 5'- GGGGTACCATGGAGGAAGTATCTCCGGC -3' |
| ABI1-nLUC-*Sal*I R | 5'- ACGCGTCGACGTTCAAGGGTTTGCTCTTGAGTT -3' |
| RRP1-cLUC-*Kpn*I F | 5'- GGGGTACCATGGATCGGAGGAGGGCGG -3' |
| RRP1-cLUC-*Bam*HI R | 5'- CGGGATCCCTATGAATCAGCATCGAGATCGTG -3' |

**Supplementary Table 2.** Off-target analysis of T1 and T2 CRISPR/Cas9 targets used in this study.

| **Target sites** | **Potential off-target sites** | **Accession numbers** | **Mis-**  **matches**  **(≤4)** | **Off-target or not** |
| --- | --- | --- | --- | --- |
| T1:GGTGCTGCTATTGCTTCCGG | GcTGCTGCTATTGCTatCGG | [AT3G28345](https://www.arabidopsis.org/servlets/TairObject?type=locus&name=AT3G28345) | 3 | **no** |
|  | tGTGCTtCaATcGCTTCCGG | AT3G07350 | 4 | **no** |
|  | aGTGCTGCTATgGCTTCtGa | AT4G34860 | 4 | **no** |
|  | GtTGCaGCcATTGtTTCCGG | AT5G08300 | 4 | **no** |
|  | GGTGCTGtcATTttTTCCGG | AT5G51500 | 4 | **no** |
|  | GGTGtTGCTATTaCTggCGG | AT5G58090 | 4 | **no** |
|  | GGTGgTGCTcTTGgTTCtGG | AT2G01690 | 4 | **no** |
|  | GGTtCTGtTAgTGCTgCCGG | AT2G29200 | 4 | **no** |
|  | GGTGCTGCTgTTGgTggCGG | AT2G39020 | 4 | **no** |
| T2:GGGAGACGGCGATCTAGTTC | none | none | none | **none** |

The lowercase letters represent the different bases between off-target and target sites.

**
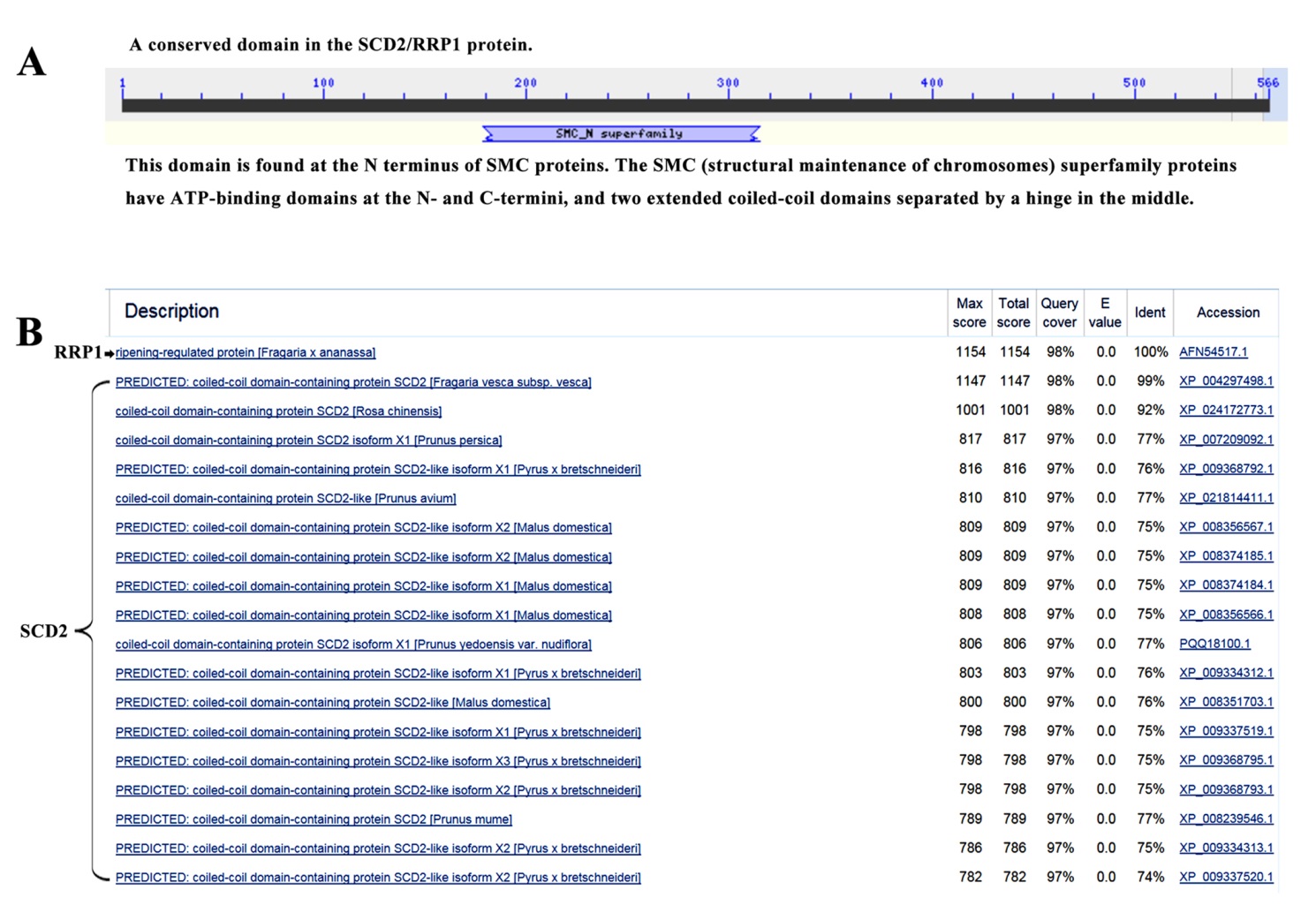
**

**Supplementary Figure 1.** [Bioinformatics](http://dict.youdao.com/w/bioinformatics/) [analysis](http://dict.youdao.com/w/analysis/) of the SCD2/RRP1.

1. The conserved domain the SCD2/RRP1; (B) Only RRP1 and SCD2 were annotated in the most homologue hits.
